# Competing risk nomogram and risk classification system for evaluating overall and cancer-specific survival in neuroendocrine carcinoma of the cervix: a population-based retrospective study

**DOI:** 10.1007/s40618-023-02261-7

**Published:** 2024-01-03

**Authors:** J. Liu, Y. Lyu, Y. He, J. Ge, W. Zou, S. Liu, H. Yang, J. Li, K. Jiang

**Affiliations:** 1 School of Basic Medical Sciences, Fourth Military Medical University, Xi’an, 710032 Shaanxi China; 2 Department of Obstetrics and Gynecology, Xijing Hospital of Fourth Military Medical University, Xi’an, 710032 Shaanxi China; 3 Department of Spine Surgery, Honghui Hospital, Xi’an Jiaotong University, Xi’an, 710054 Shaanxi China

**Keywords:** Neuroendocrine carcinoma of the cervix, Overall survival, Cancer-specific survival, Risk stratification, Competing risk, Nomogram

## Abstract

**Objective:**

Neuroendocrine carcinoma of the cervix (NECC) is a rare malignancy with poor clinical prognosis due to limited therapeutic options. This study aimed to establish a risk-stratification score and nomogram models to predict prognosis in NECC patients.

**Methods:**

Data on individuals diagnosed with NECC between 2000 and 2019 were retrieved from the Surveillance Epidemiology and End Results (SEER) database and then randomly classified into training and validation cohorts (7:3). Univariate and multivariate Cox regression analyses evaluated independent indicators of prognosis. Least absolute shrinkage and selection operator (LASSO) regression analysis further assisted in confirming candidate variables. Based on these factors, cancer-specific survival (CSS) and overall survival (OS) nomograms that predict survival over 1, 3, and 5 years were constructed. The receiver operating characteristic (ROC) curve, the concordance index (C-index), and the calibration curve estimated the precision and discriminability of the competing risk nomogram for both cohorts. Finally, we assessed the clinical value of the nomograms using decision curve analysis (DCA).

**Results:**

Data from 2348 patients were obtained from the SEER database. Age, tumor stage, T stage, N stage, chemotherapy, radiotherapy, and surgery predicted OS. Additionally, histological type was another standalone indicator of CSS prognosis. For predicting CSS, the C-index was 0.751 (95% CI 0.731 ~ 0.770) and 0.740 (95% CI 0.710 ~ 0.770) for the training and validation cohorts, respectively. Furthermore, the C-index in OS prediction was 0.757 (95% CI 0.738 ~ 0.776) and 0.747 (95% CI 0.718 ~ 0.776) for both cohorts. The proposed model had an excellent discriminative ability. Good accuracy and discriminability were also demonstrated using the AUC and calibration curves. Additionally, DCA demonstrated the high clinical potential of the nomograms for CSS and OS prediction. We constructed a corresponding risk classification system using nomogram scores. For the whole cohort, the median CSS times for the low-, moderate-, and high-risk groups were 59.3, 19.5, and 7.4 months, respectively.

**Conclusion:**

New competing risk nomograms and a risk classification system were successfully developed to predict the 1-, 3-, and 5-year CSS and OS of NECC patients. The models are internally accurate and reliable and may guide clinicians toward better clinical decisions and the development of personalized treatment plans.

**Supplementary Information:**

The online version contains supplementary material available at 10.1007/s40618-023-02261-7.

## Introduction

Neuroendocrine carcinoma of the cervix (NECC) is an uncommon histological variant of cervical cancer that has a poor prognosis, is highly invasive, and accounts for 1–1.5% of all cervical cancers [1–3]. In 2014, the World Health Organization (WHO) classified NECC into well-differentiated NECC (carcinoid and atypical carcinoid) and poorly differentiated NECC (small cell or large cell carcinoma)[4]. The majority of NECC cases are small-cell NECC (80%), followed by large-cell NECC (12%), along with other histological types that account for the remaining 8% of cases. Approximately 80% of small cell NECC involves high-risk human papillomavirus (HPV), primarily due to the HPV 18 genetic variant [2].

The biological characteristics of NECC distinguish it from squamous carcinoma or adenocarcinoma of the cervix due to its exceptionally aggressive nature, marked by a pronounced inclination for lymphatic and hematogenous dissemination [1, 5–7]. Local and distant metastases are more common in NECC than in other pathological types of cervical cancer. The brain, bone, lungs, and liver are often affected by distant metastases [8–10]. Additionally, regardless of stage, women with NECC have a much lower survival rate than those with squamous cell carcinomas[11–13].

Currently, clinical staging systems adopted by the American Joint Committee on Cancer (AJCC) and the International Federation of Obstetrics and Gynecology Staging Guidelines (FIGO) are used to determine the prognosis of cervical cancer patients. However, these staging systems are not sufficiently comprehensive to predict prognosis for patients with NECC. The 5-year survival rate varies from 30–46% in the early stages to 0–15% in the advanced stages [8]. In recent studies, the 5-year CSS and OS rates of NECC patients were 36.6% and 30.6%, respectively[8]. Given these limitations, it is necessary to construct a more comprehensive and customized prognostic model for NECC patients.

In recent years, nomograms have been used to predict the prognosis of patients with a variety of malignancies [14, 15]. Nomograms are essential in medical research for risk assessment, fostering integrated biological and clinical models. They empower personalized medicine and inform precise clinical decision-making. A nomogram is used to determine a specific clinical endpoint based on various clinical and demographic factors. Unfortunately, few prognostic nomograms and models have been designed for NECC patients to date. In this study, we utilized the nationally recognized Surveillance, Epidemiology, and End Results (SEER) database to investigate significant risk factors and develop prognostic models for CSS and OS outcomes in NECC patients.

## Materials and methods

### Study subjects and data retrieval

The Surveillance, Epidemiology, and End Results (SEER) database, encompassing data on cancer diagnoses, patient demographics (age, race, marital status, etc.), tumor characteristics (grade, histology type, TNM stage, etc.), treatment modalities (surgery, radiotherapy, chemotherapy, etc.), and survival records, covers nearly 30% of the U.S. population, making it a valuable resource for cancer-related clinical research. We accessed data from the SEER database (version 8.4.0.1) of the National Cancer Institute, spanning the years 2000 to 2019, to compile information on cervical cancer patients. A total of 2348 patients were included and comprised individuals who were diagnosed with cervical cancer (C53.0-C53.9) [histologic type ICD-O-3 = 8012–8014 (large cell carcinoma), 8041–8044 (small cell carcinoma), 8246 (neuroendocrine carcinoma), and 8045 (combined small cell carcinoma)]. The inclusion criteria were as follows: 1) age > 18 years and 2) a pathological diagnosis of cervical cancer between 2000 and 2019. However, patients were excluded if cervical cancer was not the primary cancer, if they were unaware of their surgical history, if their positive histology was unavailable or unknown, and if their survival duration lasted less than a month or information on this was unavailable.

Cervical cancer-specific death and other causes-specific death were defined as two competing events. First, the cumulative incidence function (CIF) was applied to show the probability of cervical cancer-related mortality and other cause-related mortality, and Gray’s test was applied to analyze the differences between groups.

**Fig. 1:**
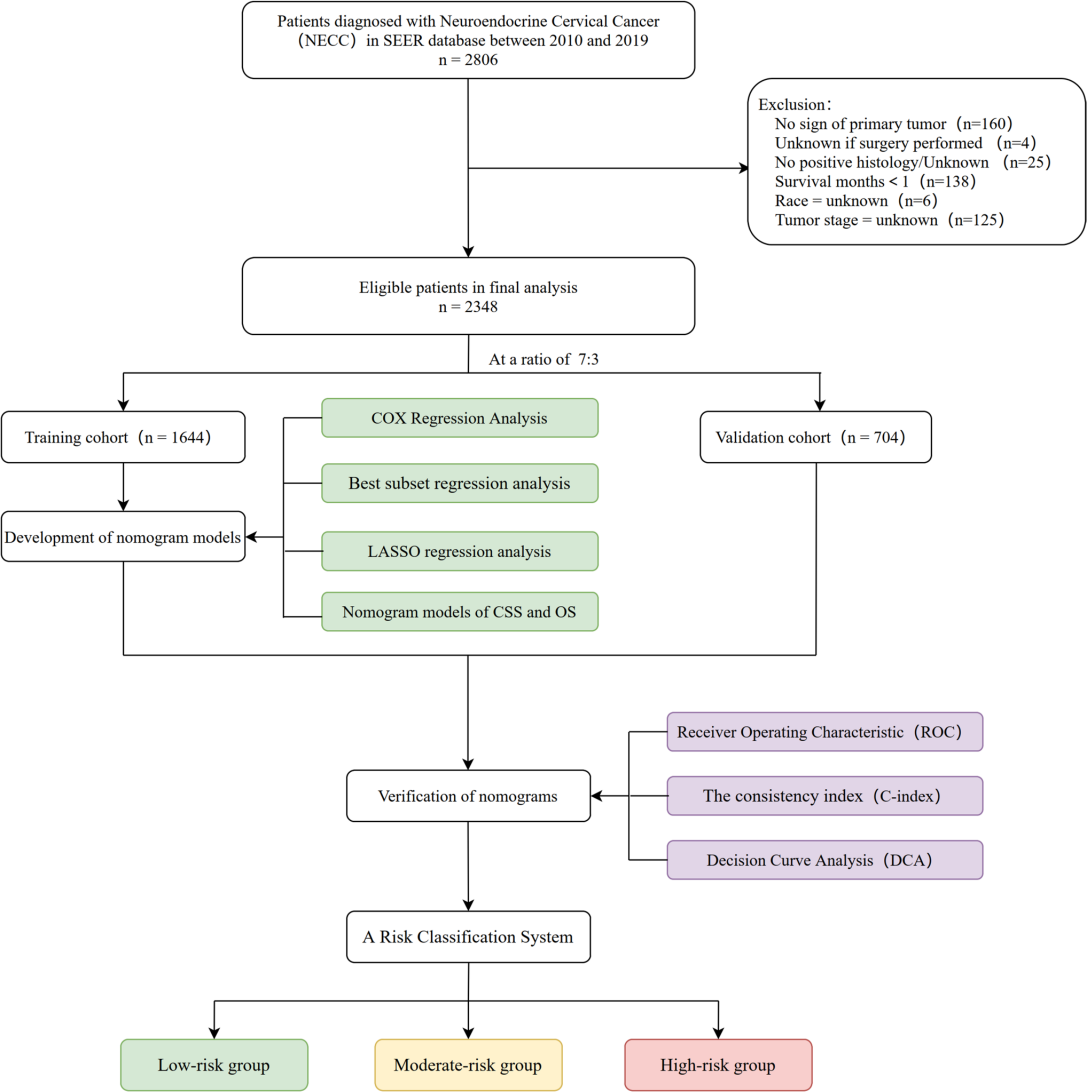
The flowchart of the study design and patient selection

The following definitions were employed at the outset of this study: cancer-specific survival (CSS) referred to cancer-related deaths, excluding noncancer fatalities, within a specified time frame; overall survival (OS) accounted for all-cause mortality during the same period, offering a comprehensive assessment of patient outcomes. Subsequently, we applied a 7:3 ratio for CSS and OS classification, randomly dividing all eligible patients into training (n = 1644) and validation cohorts (n = 704). A detailed flowchart providing an overview of the patient selection process and model development is depicted in Fig. 1. The ethics committee of Xijing Hospital (The Fourth Military Medical University) provided authorization to conduct this research. Data retrieved utilizing the SEER database is accessible without requiring patient consent. This study followed the Transparent Reporting of a Multivariable Prediction Model for Individual Prognosis or Diagnosis (TRIPOD) study reporting guidelines.

### Development and validation of the nomograms

Univariate and multivariate Cox proportional hazard regression models were used to identify potential prognostic factors for survival outcomes. Then, best subset regression (BSR) and LASSO regression were applied to select combinations of potential predictors related to CSS and OS in all NECC patients. BSR is a model selection approach that involves testing all possible combinations of predictor variables and subsequently selecting the optimal model based on specific statistical criteria. LASSO regression, introduced by Tibshirani[16], is an effective classification technique that is employed in machine-learning classification and regression studies and consistently yields impressive predictive outcomes.

Nomograms were developed utilizing LASSO regression and the BSR model to predict 1-, 3-, and 5-year OS. In contrast with BSR, which utilized a BIC minimum, when LASSO regression was applied to estimate the penalty coefficient (lambda, λ), the least mean square error (MSE) was used along with a standard error (SE). LASSO screening variables performed better than BSR screening variables in terms of net reclassification improvement (NRI) and the integrated discrimination index (IDI). Thus, the nomograms were constructed using the LASSO regression model.

The nomogram was used to develop a risk classification system relying on linear predictors that classified NECC patients into low-, moderate- and high-risk groups. X-tile software (Version 3.6.1; Robert, MD) was utilized to identify cutoff values, which were further verified in the validation cohorts and all patients. The log-rank test was utilized to compare the survival estimation between varied prediction features as calculated via Kaplan‒Meier (KM) curves and illustrated in Fig. S1. The consistency index (C-index), ROC curve, and calibration curves were utilized to assess the nomogram model's validation and predictive accuracy. Decision curve analysis (DCA) was also performed using the nomogram to test its consistency with actual observations and predictions for 1-, 3-, and 5-year survival probabilities.

### Statistical analysis

Categorical variables are represented using numbers (N) and percentages (%), whereas means and standard deviations (SD) represent continuous variables. The Chi-square test was used to analyze categorical variables, while the t test was applied for continuous variables. The Kaplan‒Meier (K‒M) method was applied to estimate OS and CSS, while log-rank tests were performed to identify significant differences. A p-value < 0.05 indicated statistical significance and statistical analyses were performed in R (v 4.1.0) and Free Statistics software (v 1.7.1).

## Results

### Demographic and clinical characteristics

A total of 2,806 patients with NECC were identified from 2000 to 2019 in the SEER database. After excluding 485 patients, 2348 patients were retained (Fig. 1). Only patients who were hospitalized for treatment after being diagnosed with NECC between 2009 and 2020 (mean age at diagnosis: 48.47 ± 15.34 years) were included in this study. Most patients (71.9%) were white, and 64.2% were diagnosed with small cell NECC (Table 1).

**Table 1:** Patients characteristics in the training and validation cohort

Characteristics	Whole chort (n = 2348)	Training cohort (n = 1644)	Validation cohort (n = 704)	P-value
Year of diagnosis				0.935
2000–2009	1147 (48.9)	804 (48.9)	343 (48.7)	
2010–2019	1201 (51.1)	840 (51.1)	361 (51.3)	
Age, Mean ± SD	48.5 ± 15.3	48.6 ± 15.4	48.2 ± 15.2	0.542
Race				0.964
White	1689 (71.9)	1184 (72.0)	505 (71.7)	
Black	329 (14.0)	231 (14.1)	98 (13.9)	
Other	330 (14.1)	229 (13.9)	101 (14.3)	
Marital status				0.650
Married	1022 (43.5)	702 (42.7)	320 (45.5)	
Divorced/widowed/separated	545 (23.2)	390 (23.7)	155 (22.0)	
Single	682 (29.0)	482 (29.3)	200 (28.4)	
Unknown	99 (4.2)	70 (4.3)	29 (4.1)	
Histology				0.076
Large cell carcinoma	231 (9.80)	150 (9.10)	81 (11.5)	
Small cell carcinoma	1507 (64.2)	1077 (65.5)	430 (61.1)	
Other	610 (26.0)	417 (25.4)	193 (27.4)	
Mixed				0.472
No	2255 (96.0)	1582 (96.2)	673 (95.6)	
Yes	93 (4.0)	62 (3.8)	31 (4.4)	
Grade				0.093
Well/Moderately differentiated	34 (1.4)	22 (1.3)	12 (1.7)	
Poorly or undifferentiated	1574 (67.0)	1082 (65.8)	492 (69.9)	
Unknown	740 (31.5)	540 (32.8)	200 (28.4)	
Tumor stage				0.938
Localized	607 (25.9)	424 (25.8)	183 (26.0)	
Regional	833 (35.5)	587 (35.7)	246 (34.9)	
Distant	908 (38.7)	633 (38.5)	275 (39.1)	
T stage				0.51
T1	799 (34.0)	552 (33.6)	247 (35.1)	
T2	435 (18.5)	320 (19.5)	115 (16.3)	
T3	392 (16.7)	273 (16.6)	119 (16.9)	
T4	75 (3.2)	51 (3.1)	24 (3.4)	
Tx	647 (27.6)	448 (27.3)	199 (28.3)	
N stage				0.286
N0	899 (38.3)	624 (38)	275 (39.1)	
N1	777 (33.1)	560 (34.1)	217 (30.8)	
Unknown	672 (28.6)	460 (28)	212 (30.1)	
M stage				0.478
M0	1230 (52.4)	861 (52.4)	369 (52.4)	
M1	714 (30.4)	509 (31.0)	205 (29.1)	
Unknown	404 (17.2)	274 (16.7)	130 (18.5)	
Surgery or not				0.65
No	1284 (54.7)	894 (54.4)	390 (55.4)	
Yes	1064 (45.3)	750 (45.6)	314 (44.6)	
Tumor size				0.489
< 4 cm	623 (26.5)	432 (26.3)	191 (27.1)	
≥ 4 cm	997 (42.5)	711 (43.2)	286 (40.6)	
Unknown	728 (31.0)	501 (30.5)	227 (32.2)	
Radiotherapy				0.201
None/Unknown	920 (39.2)	658 (40.0)	262 (37.2)	
Yes	1428 (60.8)	986 (60.0)	442 (62.8)	
Chemotherapy				0.784
None/Unknown	418 (17.8)	295 (17.9)	123 (17.5)	
Yes	1930 (82.2)	1349 (82.1)	581 (82.5)	
Vital status				0.865
Live	773 (32.9)	543 (33.0)	230 (32.7)	
Dead	1575 (67.1)	1101 (67.0)	474 (67.3)	
Survival months, Mean ± SD	38.2 ± 52.0	38.7 ± 52.5	37.1 ± 50.9	0.488

**Fig. 2:**
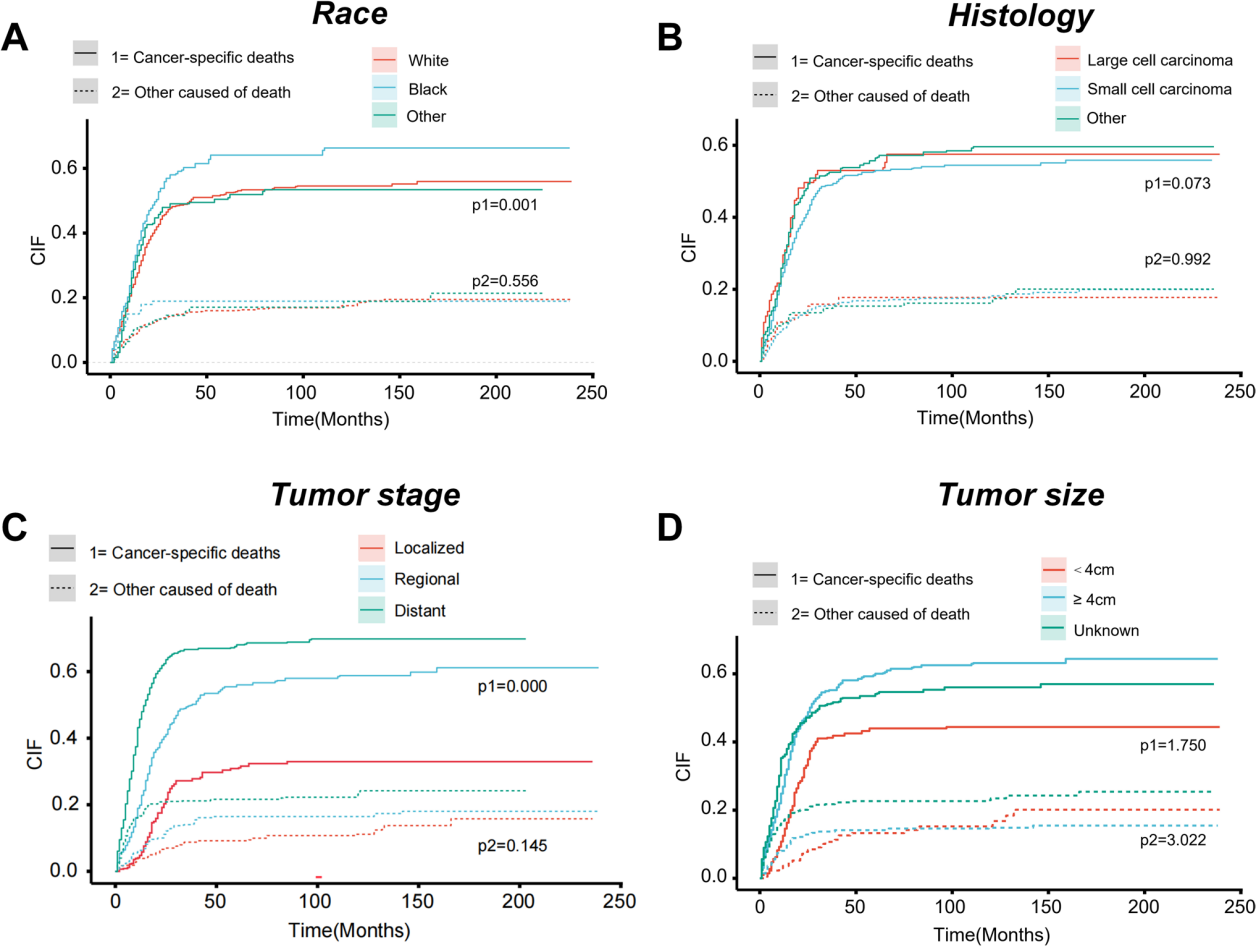
CIF curves for NECC by different variates. (A)Race; (B)Histology; (C) Tumor stage; (D) Tumor size. P values were calculated with the Fine-Gray test. p1 represents the difference of cancer-specific deaths. p2 represents the difference of other cause of death

There were no statistically significant differences (variations) (P > 0.05) between the training (n = 1644) and validation (n = 704) cohorts. In the training cohort, 1184 (72.0%) patients were white, 1077 (65.5%) patients had small cell carcinoma, and 633 patients (38.5%) developed distant metastasis. In terms of disease staging, 750 (45.6%) patients underwent surgical treatment, 986 (60.0%) underwent radiation therapy, and 1349 (82.1%) received chemotherapy. The median duration of follow-up for the entire cohort was maintained at 16 months. The cumulative incidences of cervical cancer-specific mortality and other causes of mortality are presented in Fig. 2.

### Predictor screening

**Fig. 3:**
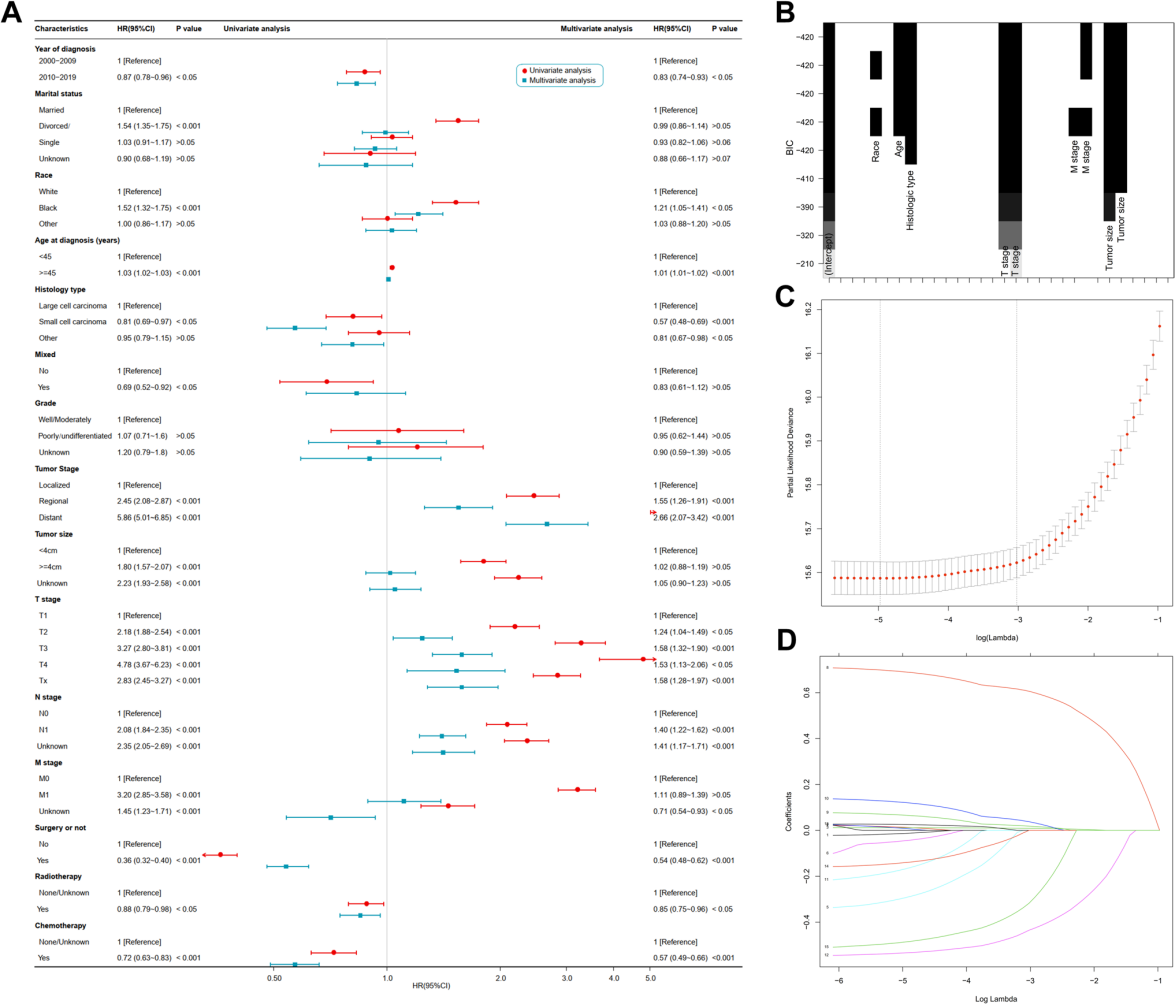
Three methods of screening predictors. Univariate and multivariate cox regression of CSS forest plot (A), best subset regression (BSR) (B), and LASSO regression (C, D). (LASSO coefficients 1. Age 2.T stage 3. N stage 4. Surgery or not 5. Chemotherapy 6. Tumor stage)

Data from a total of 2348 patients were included in this retrospective analysis, of whom 1575 (67.1%) passed away. The cancer-specific mortality rate for NECCs was 62.0% compared to 5.1% for other causes. Univariate and multivariate Cox proportional hazard regression analyses were conducted to investigate prognostic factors for CSS and OS. Factors such as the year of diagnosis, age, histology type, tumor stage, T stage, N stage, whether surgery was performed or not, radiotherapy, and chemotherapy were all significantly (P < 0.05) associated with wither CSS or OS (Fig. 3a, Figure S2, Tables S1 and S2).

**Fig. 4:**
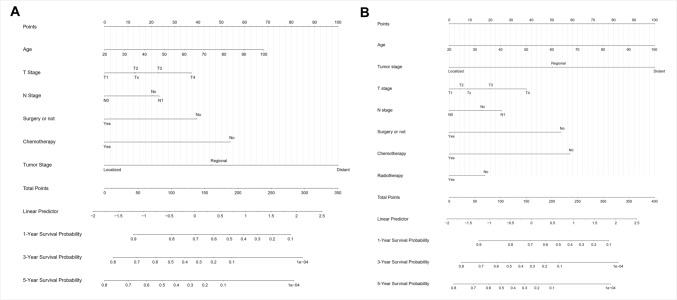
Nomograms for predicting the 1-, 3- and 5-year CSS (A) and OS (B) in NECC patients

As per the BSR model, seven factors (age, race, histologic type, T-, N-, & M- stages, and tumor size) were selected for developing nomograms that predict the 1-, 3- and 5-year CSS as well as OS (Figs. 3b–d). By comparing NRI and IDI, it was found that the prediction model constructed using LASSO screening variables outperformed BSR screening variables (Table S3). The results of the LASSO regression analysis for NECC patients are illustrated in Fig. 3b, c. Finally, six factors [age, T- and N- stages, surgery (Y/N) and radiotherapy] and seven factors (age, T- and N- stages, surgery (Y/N), radiotherapy, and chemotherapy) were chosen to develop nomograms to predict 1-, 3- and 5-year CSS and OS, respectively (Fig. 4).

### Development and validation of prognostic nomograms for OS and CSS

Based on the predictive nomogram for CSS, the training and validation cohort C-index values were 0.751 (95% CI 0.731–0.770) and 0.740 (95% CI 0.710–0.770), respectively. The C-index for OS from the prognostic nomogram was 0.757 (95% CI 0.738–0.776) for the training cohort and 0.747 (95% CI 0.718–0.776) for the validation cohort (Table S4).

For the training set, the nomogram’s C-index was 0.757 (95% CI 0.738–0.776), whereas a C-index of 0.697 (95% CI 0.678–0.715) was recorded for the AJCC system (TNM stage) for OS. These similar findings helped to compare both nomograms and the AJCC system across training and validation cohorts in terms of CSS. This revealed a better prediction effect of the nomogram than the AJCC system on comparing the NRI, IDI, and C-index (Tables S1, S2).

**Fig. 5:**
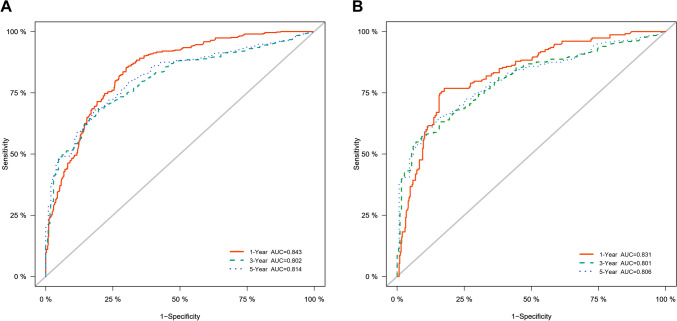
Validation of the nomogram model in training (A) and validation cohorts (B) using 1-, 3-, and 5-Year ROC curves

**Fig. 6:**
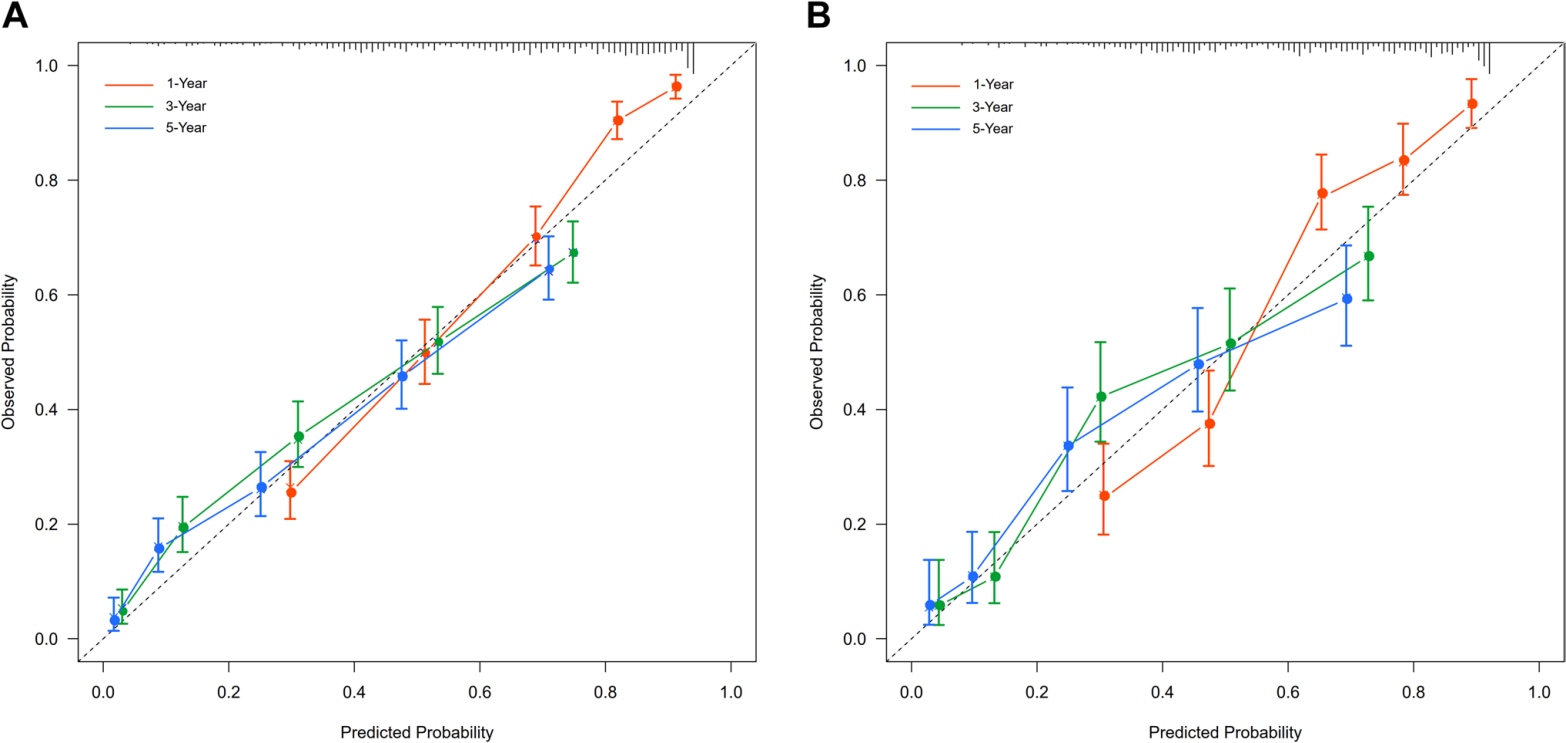
Calibration plot diagrams of CSS-associated nomograms from training and validation cohorts. Calibration curves for 1-, 3-, and 5-year CSS in the training (A) and the validation cohorts (B)

According to the ROC curves for prognosis predictive nomograms, the 1-, 3- and 5-year AUCs for the CSS nomogram were 0.843, 0.802, and 0.814, respectively, in the training cohort and 0.831, 0.801, and 0.806, respectively, in the validation cohort (Figs. 5A, 5B). Concurrently, the 1-, 3- and 5-year AUCs for the OS nomogram in the training cohort were 0.847, 0.807, and 0.819, respectively, and 0.837, 0.800, and 0.804, respectively, in the validation cohort (Figs. 5C, 5D). The calibration plots for the CSS-associated nomogram for 1-, 3-, and 5-year CSS in the training (A) and validation cohorts (B) were perfectly fitted and close to the ideal line (45°) (Fig. 6). According to the results, the prognostic nomograms achieved outstanding performance in distinguishing CSS from OS and in predicting outcomes.

**Fig. 7:**
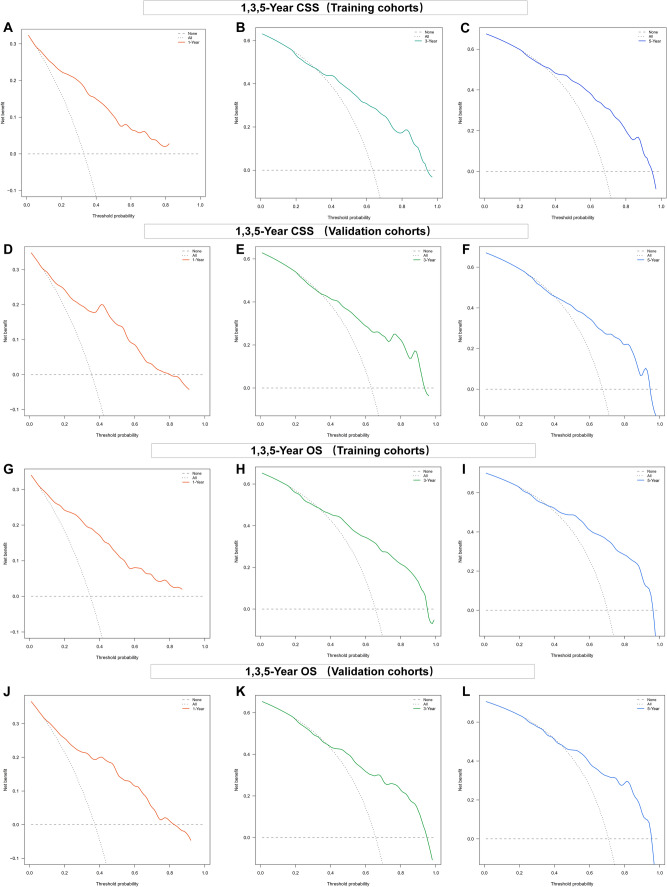
Decision curves for predicting 1-year CSS nomogram (A); 3-year CSS nomogram (B); 5-year CSS nomogram in training cohorts (C); 1-year CSS nomogram (D); 3-year CSS nomogram (E); 5-year CSS nomogram in validation cohorts (F). Decision curves for 1-, 3- and 5-year OS in the training (G, H, I) and the validation cohorts (J, K, L)

Consistently positive net clinical benefits were estimated for patients using DCA among a broad range of probability thresholds for CSS and OS prediction curves over 1, 3, and 5 years (Fig. 7). These novel nomogram models showed significant clinical utility in predicting the survival of NECC patients.

### Risk classification system

Risk scores (linear predictors) were then determined using the CSS nomogram model, and a risk classification system was developed by categorizing patients into low-, moderate-, and high-risk groups. A cutoff analysis by the X-title program was conducted for the training group (Fig. S1).

**Fig. 8:**
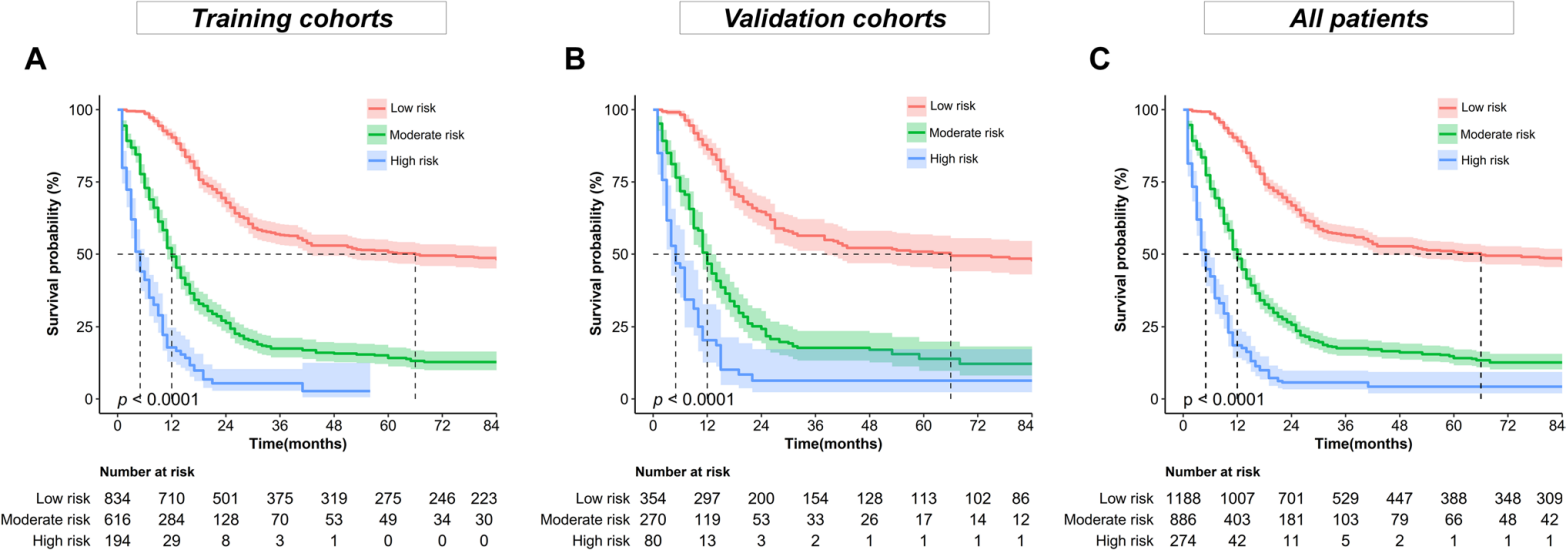
Comparing CSS in the low-risk, moderate–risk, and high-risk groups in the (A) training cohorts, (B) validation cohorts, and (C) all patients

In the training group, low-, moderate-, and high-risk NECC patients had median CSS times of 60.2, 19.6, and 6.8 months, respectively, whereas the 1-year CSS rates in these risk groups were 90.4, 49.2, and 16.6%, respectively. The median CSS times for low-, moderate-, and high-risk patients in the validation group were 57.2 months, 19.1 months, and 8.8 months, respectively, whereas the 1-year OS rates for the three groups were 86.3%, 46.7%, and 20.3%, respectively. For the whole cohort, the median CSS times for the low-, moderate-, and high-risk groups were 59.3, 19.5, and 7.4 months, respectively (all P < 0.0001). The 1-year OS rates in the low-, moderate- and high-risk groups were 89.1%, 48.4%, and 17.7%, respectively (Fig. 8).

## Discussion

NECC is an uncommon, highly invasive, pathological type of cervical cancer with a poor prognosis. Although most cases of cervical cancer are diagnosed at an early stage, NECC is more aggressive than other subtypes, accounting for 1%-2% of all cervical cancer cases. A retrospective analysis of data over 25 years performed by Gibbs et al. revealed an increase in the incidence of NECC tumors, but no improvement has been made in patient OS. This study utilized the SEER database and found that only 36.4% of NECC patients were diagnosed at an early stage [17, 18]. It has been estimated that NECC patients have a mortality rate 1.8 times greater than that of women with cervical SCC[19]. According to a previous study, the 5-year CSS for patients at stages I-IIA, IIB­IVA, and IVB were 36.8%, 8.9%, and 0%, respectively[3]. Due to the rarity of NECC, many challenges and uncertainties preclude the establishment of an effective treatment management plan for NECC patients. The results of the current study are consistent with previous findings in that small-cell NECC is the most common pathological subtype. The prognosis for small-cell NECC is poor, with a 40-month mean survival rate and a 34% five-year survival rate[13]. Margolis et al. published a study with the largest cohort of women with NECC to date[7]. In total, 1896 NECC patients were identified utilizing the National Cancer Database (NCDB). Multivariate regression analysis revealed that NECC patients at all clinical stages possess a considerably higher risk of death than SCC patients.

Clinical staging of cervical cancer is mainly performed by both the AJCC and FIGO. However, there are other critical factors affecting the prognosis of NECC patients that cannot be adequately explained by these two clinical staging systems. Even patients with similar clinical stages may have different prognostic outcomes due to various factors. Given the lack of research on predicting CSS and OS in NECC patients, it is essential to identify crucial characteristics that impact the prognosis of NECC patients.

Univariate and multivariate Cox proportional hazard regression analyses were conducted to investigate prognostic factors for CSS as well as OS in NECC patients whose data were obtained from the SEER database. The outcomes from this study demonstrate that age, race, histological type, T and N stages, tumor stage, surgery (Y/N), radiotherapy, and chemotherapy are independent prognostic characteristics for NECC in terms of CSS and OS. Based on these results, variables linked to the NECC patient prognosis were screened utilizing BSR and LASSO regression, and novel nomogram models were constructed. In a previous study that utilized the SEER database, a prognostic model was developed for predicting OS in small-cell carcinoma of the cervix[15]. Considering the heterogeneity of pathology, more pathologies, such as large cell neuroendocrine carcinoma and other types, were included in this study. Moreover, as OS may overestimate the survival of NECC, a CSS predictive model was constructed to account for patient mortality due to tumors. To our knowledge, this is the first study to develop and validate novel nomograms for predicting CSS and OS in NECC patients.

The presence of tumor metastases is a key independent prognostic factor in NECC, according to a previously published retrospective study[20]. In addition, another study revealed that patients with distant metastases can live longer after surgery compared to nonsurgical patients[18]. In line with previous studies, the results of this study consistently reflect the relationship between tumor metastasis and CSS or OS. According to previous research, a greater proportion of patients who had their first major surgery survived compared to those who had direct radiation[21]. Reportedly, patients with small-cell cervical cancer benefited from the platinum-based combination as an independent indicator of prognosis for improving survival status[22–24]. The findings of the current study are likewise consistent with those of these studies.

Recently, two studies evaluating NECC nomograms identified the importance of pathological characteristics in the construction of prediction models, while information related to treatment variables remained notably insufficient[25, 26]. In this study, our nomograms included additional information regarding age, tumor characteristics, and treatment choices, which may minimize bias. The nomograms developed in this study had sufficient discriminatory power, as evidenced by C-index values of 0.751 and 0.757, to predict OS and CSS, respectively. Nomograms of OS and CSS had AUCs ranging from 0.802 to 0.847 corresponding to 12, 36, and 60 months. Finally, under different threshold probabilities, DCA curves showed robust positive net benefits. The models developed herein predict NECC patient prognosis significantly better than AJCC and FIGO staging. Thus, these nomograms may provide clinicians with a more practical tool to formulate appropriate individualized treatment options for NECC patients, thereby potentially improving clinical outcomes.

Based on the linear predictors obtained from each patient, a brand-new approach for categorizing risks was created. There was a median CSS time of 65 months in the low-risk group, while less than half a year of CSS time was observed in the high-risk group. Utilizing a nomogram for risk stratification is instrumental in facilitating clinical practitioners to accurately evaluate prognosis and to devise personalized treatment strategies. For instance, when dealing with high-risk patients, careful deliberation should be given to incorporating multimodal therapies that encompass radiotherapy, chemotherapy, targeted therapy, and immunotherapy, as the core initial treatment approach to improve clinical prognosis. According to these data, patients at high risk for NECC should consider aggressive combination therapy to improve their survival outcomes and quality of life. As shown by Lu et al., systemic therapy, which includes surgery combined with chemoradiotherapy, is the most effective therapy for early-stage NECC patients[2]. Apart from surgery combined with chemoradiotherapy, the application of combined immunotherapies has also shown promising results in this area. For example, an immunotherapy regimen involving a combination of ipilimumab/nivolumab was found to affect responses in clinical trials and case studies in recurrent NECC[27–29]. Recently, Paterniti et al. reported on a patient with NECC who responded completely to this drug combination[30]. To improve the survival rate of NECC patients, a multimodal therapeutic approach must be explored and individualized according to the patient's requirements.

Several limitations are present in this study. First, our study used a retrospective design and analyzed data from the SEER database. Second, some clinical information was unavailable, such as HPV infection status, comorbidities, radiotherapy dose, specific chemotherapy information, and subsequent treatment options for metastatic sites. Third, new therapeutic approaches, such as immune checkpoint inhibitors (ICIs) and targeted drugs, were not included in the SEER database. Thus, some clinical factors that could influence prognosis were not incorporated into the constructed models. Finally, these nomograms and risk classification systems were developed using data collected only in the United States. Thus, caution should be taken when applying our approach to different countries or populations. Large prospective studies should be conducted to validate the results of this study.

## Conclusions

Novel nomograms were constructed that effectively predict 1-, 3-, and 5-year CSS and OS in NECC patients using a combination of four clinicopathological features and three treatment-related parameters obtained from the SEER database. External verification also revealed that performance was satisfactory. In addition, a new risk classification system was established that effectively divides NECC patients into three distinct risk categories. The models presented in the current work have demonstrated in our retrospective analysis to be potentially useful to predict the survival of NECC patients. Further larger and prospective studies are needed to validate the achieved results, eventually leading to the use of these nomograms models in the clinical practice.

### Supplementary Information

Below is the link to the electronic supplementary material.

Supplementary file1 (DOCX 847 kb)

## Data Availability

The SEER database, which is downloadable at www.seer.cancer.gov, served as the source of all the data used in this research.

## Abbreviations

SEER: Surveillance, Epidemiology, and End Results
LASSO: Least absolute shrinkage and selection operator
ICD-O-3: International Classification of Diseases for Oncology, Third Edition
AJCC: American Joint Committee on Cancer
SD: Standard deviation
IQR: Interquartile range
C-index: Concordance index
DCA: Decision curve analysis
