# Supplementary material for: Competing risk nomogram and risk classification system for evaluating overall and cancer-specific survival in neuroendocrine carcinoma of the cervix: a population-based retrospective study
Source: J Endocrinol Invest. 2024 Jan 3;47(6):1545–57. doi: 10.1007/s40618-023-02261-7 (PMC11143030; doi:10.1007/s40618-023-02261-7)
Supplement: Supplementary file 1 — Supplementary file1 (DOCX 847 kb) [file 40618_2023_2261_MOESM1_ESM.docx]

Supplementary Material

**Competing risk nomogram and risk classification system for evaluating overall and cancer-specific survival in neuroendocrine cervical cancer: a population-based retrospective study**

**Jia-qi Liu^2†^, Yan-hong Lyu^1†^, Yuan-yuan^1†^ He, Jun-li Ge^1^, Wei Zou^1^, Shu-juan Liu^1^, Hong Yang^1^, Jia Li^1^*, Kuo Jiang^3^***

*** Correspondence:**

Corresponding Author:

Kuo Jiang, email: jiangkuo520@qq.com

Jia Li, email: lijia219@yeah.net ;

# Supplementary Data

Additional supporting information may be found online in the Supporting Information section at the end of the article.

# Supplementary Figures and Tables

## Supplementary Figures


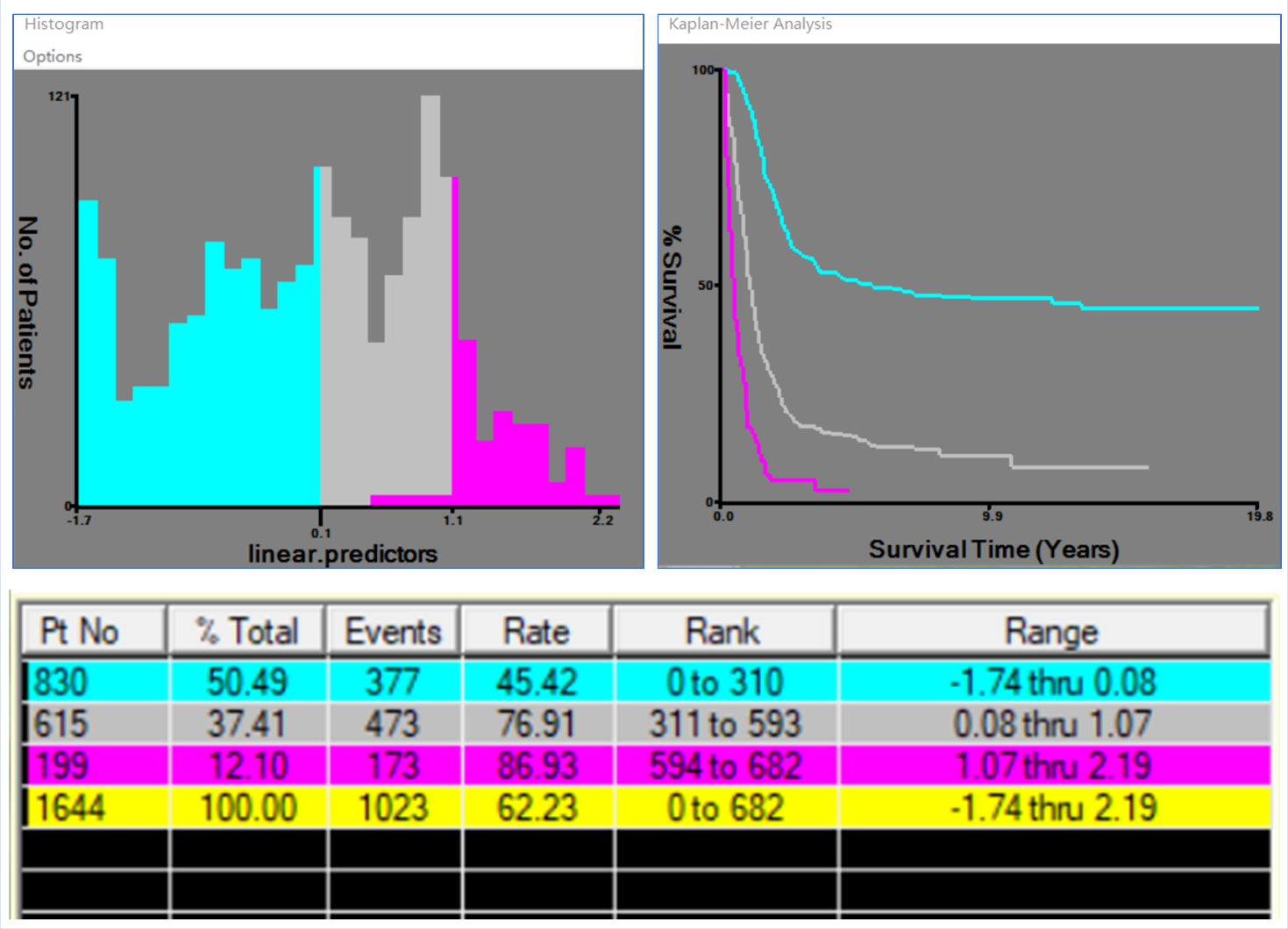
**Supplementary Figure S1.** The X-tile analyzes the cutoff value of linear predictor for the training group.

**Supplementary Figure S2.** Univariate and multivariate Cox regression of OS forest plot in NECC patients.


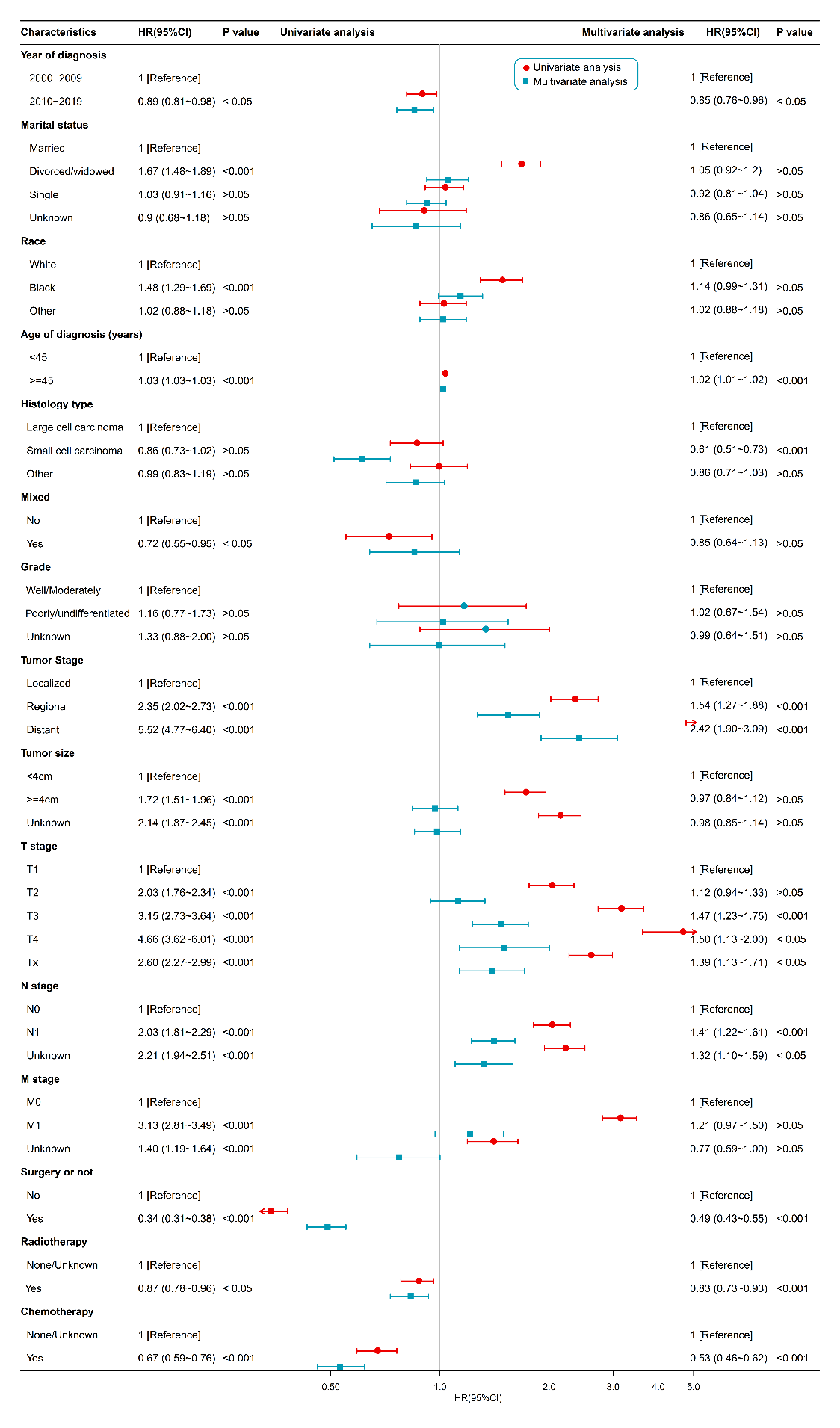


## 2.2 Supplementary Tables

**TABLE S1| Univariate and multivariate Cox regression analyses to CSS in NECC patients.**

| **Characteristics** | Univariate analysis | | Multivariate analysis | |
| --- | --- | --- | --- | --- |
|  | HR (95%CI) | P value | HR (95%CI) | P value |
| **Year of diagnosis** |  |  |  |  |
| 2000-2009 | 1 [Reference] |  | 1 [Reference] |  |
| 2010-2019 | 0.87 (0.78~0.96) | **0.007** | 0.83 (0.74~0.93) | **0.002** |
| **Marital status** |  |  |  |  |
| Married | 1 [Reference] |  | 1 [Reference] |  |
| Divorced/widowed/separated | 1.54 (1.35~1.75) | **< 0.001** | 0.99 (0.86~1.14) | 0.882 |
| Single | 1.03 (0.91~1.17) | 0.593 | 0.93 (0.82~1.06) | 0.278 |
| Unknown | 0.90 (0.68~1.19) | 0.458 | 0.88 (0.66~1.17) | 0.374 |
| **Race** |  |  |  |  |
| White | 1 [Reference] |  | 1 [Reference] |  |
| Black | 1.52 (1.32~1.75) | **< 0.001** | 1.21 (1.05~1.41) | **0.010** |
| Other | 1.00 (0.86~1.17) | 0.962 | 1.03 (0.88~1.20) | 0.972 |
| **Age at diagnosis (years)** |  |  |  |  |
| ＜45 | 1 [Reference] |  | 1 [Reference] |  |
| ≥45 | 1.03 (1.02~1.03) | **< 0.001** | 1.01 (1.01~1.02) | **<0.001** |
| **Histology type** |  |  |  |  |
| Large cell carcinoma | 1 [Reference] |  | 1 [Reference] |  |
| Small cell carcinoma | 0.81 (0.69~0.97) | **0.019** | 0.57 (0.48~0.69) | **<0.001** |
| Other | 0.95 (0.79~1.15) | 0.628 | 0.81 (0.67~0.98) | **0.029** |
| **Mixed** |  |  |  |  |
| No | 1 [Reference] |  | 1 [Reference] |  |
| Yes | 0.69 (0.52~0.92) | **0.012** | 0.83 (0.61~1.12) | 0.230 |
| **Grade** |  |  |  |  |
| Well/Moderately differentiated | 1 [Reference] |  | 1 [Reference] |  |
| Poorly or undifferentiated | 1.07 (0.71~1.6) | 0.758 | 0.95 (0.62~1.44) | 0.802 |
| Unknown | 1.20 (0.79~1.8) | 0.393 | 0.90 (0.59~1.39) | 0.647 |
| **Tumor Stage** |  |  |  |  |
| Localized | 1 [Reference] |  | 1 [Reference] |  |
| Regional | 2.45 (2.08~2.87) | **<0.001** | 1.55 (1.26~1.91) | **<0.001** |
| Distant | 5.86 (5.01~6.85) | **<0.001** | 2.66 (2.07~3.42) | **<0.001** |
| **Tumor size** |  |  |  |  |
| ＜4cm | 1 [Reference] |  | 1 [Reference] |  |
| ≥4cm | 1.80 (1.57~2.07) | **< 0.001** | 1.02 (0.88~1.19) | 0.759 |
| Unknown | 2.23 (1.93~2.58) | **< 0.001** | 1.05 (0.90~1.23) | 0.543 |
| **T stage** |  |  |  |  |
| T1 | 1 [Reference] |  | 1 [Reference] |  |
| T2 | 2.18 (1.88~2.54) | **< 0.001** | 1.24 (1.04~1.49) | **0.018** |
| T3 | 3.27 (2.80~3.81) | **< 0.001** | 1.58 (1.32~1.90) | **<0.001** |
| T4 | 4.78 (3.67~6.23) | **< 0.001** | 1.53 (1.13~2.06) | **0.006** |
| Tx | 2.83 (2.45~3.27) | **< 0.001** | 1.58 (1.28~1.97) | **<0.001** |
| **N stage** |  |  |  |  |
| N0 | 1 [Reference] |  | 1 [Reference] |  |
| N1 | 2.08 (1.84~2.35) | **< 0.001** | 1.40 (1.22~1.62) | **<0.001** |
| Unknown | 2.35 (2.05~2.69) | **< 0.001** | 1.41 (1.17~1.71) | **0.001** |
| **M stage** |  |  |  |  |
| M0 | 1 [Reference] |  | 1 [Reference] |  |
| M1 | 3.20 (2.85~3.58) | **< 0.001** | 1.11 (0.89~1.39) | 0.338 |
| Unknown | 1.45 (1.23~1.71) | **< 0.001** | 0.71 (0.54~0.93) | 0.013 |
| **Surgery or not** |  |  |  |  |
| No | 1 [Reference] |  | 1 [Reference] |  |
| Yes | 0.36 (0.32~0.40) | **< 0.001** | 0.54 (0.48~0.62) | **<0.001** |
| **Radiotherapy** |  |  |  |  |
| None/Unknown | 1 [Reference] |  | 1 [Reference] |  |
| Yes | 0.88 (0.79~0.98) | **0.018** | 0.85 (0.75~0.96) | **0.011** |
| **Chemotherapy** |  |  |  |  |
| None/Unknown | 1 [Reference] |  | 1 [Reference] |  |
| Yes | 0.72 (0.63~0.83) | **< 0.001** | 0.57 (0.49~0.66) | **<0.001** |

**TABLE S2| Univariate and multivariate Cox regression analyses to OS in NECC patients.**

| **Characteristics** | Univariate analysis | | Multivariate analysis | |
| --- | --- | --- | --- | --- |
|  | HR (95%CI) | P value | HR (95%CI) | P value |
| **Year of diagnosis** |  |  |  |  |
| 2000-2009 | 1 [Reference] |  | 1 [Reference] |  |
| 2010-2019 | 0.89 (0.81~0.98) | **0.024** | 0.85 (0.76~0.96) | **0.006** |
| **Marital status** |  |  |  |  |
| Married | 1 [Reference] |  | 1 [Reference] |  |
| Divorced/widowed/separated | 1.67 (1.48~1.89) | **<0.001** | 1.05 (0.92~1.2) | 0.460 |
| Single | 1.03 (0.91~1.16) | 0.680 | 0.92 (0.81~1.04) | 0.186 |
| Unknown | 0.9 (0.68~1.18) | 0.442 | 0.86 (0.65~1.14) | 0.293 |
| **Race** |  |  |  |  |
| White | 1 [Reference] |  | 1 [Reference] |  |
| Black | 1.48 (1.29~1.69) | **< 0.001** | 1.14 (0.99~1.31) | 0.078 |
| Other | 1.02 (0.88~1.18) | 0.824 | 1.02 (0.88~1.18) | 0.827 |
| **Age of diagnosis (years)** |  |  |  |  |
| ＜45 | 1 [Reference] |  | 1 [Reference] |  |
| ≥45 | 1.03 (1.03~1.03) | **<0.001** | 1.02 (1.01~1.02) | **<0.001** |
| **Histology type** |  |  |  |  |
| Large cell carcinoma | 1 [Reference] |  | 1 [Reference] |  |
| Small cell carcinoma | 0.86 (0.73~1.02) | 0.074 | 0.61 (0.51~0.73) | **<0.001** |
| Other | 0.99 (0.83~1.19) | 0.935 | 0.86 (0.71~1.03) | 0.104 |
| **Mixed** |  |  |  |  |
| No | 1 [Reference] |  | 1 [Reference] |  |
| Yes | 0.72 (0.55~0.95) | **0.019** | 0.85 (0.64~1.13) | 0.268 |
| **Grade** |  |  |  |  |
| Well/Moderately differentiated | 1 [Reference] |  | 1 [Reference] |  |
| Poorly or undifferentiated | 1.16 (0.77~1.73) | 0.485 | 1.02 (0.67~1.54) | 0.937 |
| Unknown | 1.33 (0.88~2.00) | 0.173 | 0.99 (0.64~1.51) | 0.950 |
| **Tumor Stage** |  |  |  |  |
| Localized | 1 [Reference] |  | 1 [Reference] |  |
| Regional | 2.35 (2.02~2.73) | **<0.001** | 1.54 (1.27~1.88) | **<0.001** |
| Distant | 5.52 (4.77~6.40) | **<0.001** | 2.42 (1.90~3.09) | **<0.001** |
| **Tumor size** |  |  |  |  |
| ＜4cm | 1 [Reference] |  | 1 [Reference] |  |
| ≥4cm | 1.72 (1.51~1.96) | **<0.001** | 0.97 (0.84~1.12) | 0.642 |
| Unknown | 2.14 (1.87~2.45) | **<0.001** | 0.98 (0.85~1.14) | 0.828 |
| **T stage** |  |  |  |  |
| T1 | 1 [Reference] |  | 1 [Reference] |  |
| T2 | 2.03 (1.76~2.34) | **<0.001** | 1.12 (0.94~1.33) | 0.211 |
| T3 | 3.15 (2.73~3.64) | **<0.001** | 1.47 (1.23~1.75) | **<0.001** |
| T4 | 4.66 (3.62~6.01) | **<0.001** | 1.50 (1.13~2.00) | **0.005** |
| Tx | 2.60 (2.27~2.99) | **<0.001** | 1.39 (1.13~1.71) | **0.002** |
| **N stage** |  |  |  |  |
| N0 | 1 [Reference] |  | 1 [Reference] |  |
| N1 | 2.03 (1.81~2.29) | **<0.001** | 1.41 (1.22~1.61) | **<0.001** |
| Unknown | 2.21 (1.94~2.51) | **<0.001** | 1.32 (1.10~1.59) | **0.003** |
| **M stage** |  |  |  |  |
| M0 | 1 [Reference] |  | 1 [Reference] |  |
| M1 | 3.13 (2.81~3.49) | **<0.001** | 1.21 (0.97~1.50) | 0.085 |
| Unknown | 1.40 (1.19~1.64) | **<0.001** | 0.77 (0.59~1.00) | 0.054 |
| **Surgery or not** |  |  |  |  |
| No | 1 [Reference] |  | 1 [Reference] |  |
| Yes | 0.34 (0.31~0.38) | **<0.001** | 0.49 (0.43~0.55) | **<0.001** |
| **Radiotherapy** |  |  |  |  |
| None/Unknown | 1 [Reference] |  | 1 [Reference] |  |
| Yes | 0.87 (0.78~0.96) | **0.005** | 0.83 (0.73~0.93) | **0.001** |
| **Chemotherapy** |  |  |  |  |
| None/Unknown | 1 [Reference] |  | 1 [Reference] |  |
| Yes | 0.67 (0.59~0.76) | **<0.001** | 0.53 (0.46~0.62) | **<0.001** |

**TABLE S3 | The comparison between our nomograms, AJCC staging and BSR-based model, by IDI and NRI.**

|  | Nomogram vs AJCC | | | *P* value | Nomogram vs BSR-based model | | | *P* value |
| --- | --- | --- | --- | --- | --- | --- | --- | --- |
|  | 12-month | 36-month | 60-month |  | 12-month | 36-month | 60-month |  |
| CSS |  |  |  |  |  |  |  |  |
| IDI | 0.192 | 0.067 | 0.160 | ＜0.001 | 0.124 | 0.121 | 0.114 | ＜0.001 |
| NRI | 0.218 | 0.039 | 0.234 | ＜0.001 | 0.128 | 0.074 | 0.036 | ＜0.001 |
| OS |  |  |  |  |  |  |  |  |
| IDI | 0.215 | 0.178 | 0.168 | ＜0.001 | 0.136 | 0.132 | 0.122 | ＜0.001 |
| NRI | 0.285 | 0.050 | 0.227 | ＜0.001 | 0.280 | 0.138 | 0.068 | ＜0.001 |

**TABLE S4** | **The C-index comparison between our nomograms and the current AJCC staging system.**

| **Cohorts** | **Nomogram** | | **AJCC system** | | ***p* value** |
| --- | --- | --- | --- | --- | --- |
|  | C-index | 95%CI | C-index | 95%CI |  |
| Training cohort，CSS | 0.751 | 0.731~0.770 | 0.704 | 0.685~0.724 | ＜0.001 |
| Training cohort，OS | 0.757 | 0.738~0.776 | 0.697 | 0.678~0.715 | ＜0.001 |
| Validation cohort，CSS | 0.740 | 0.710~0.770 | 0.698 | 0.668~0.727 | ＜0.001 |
| Validation cohort，OS | 0.747 | 0.718~0.776 | 0.695 | 0.666~0.724 | ＜0.001 |
